# Detecting Depression Through Gait Data: Examining the Contribution of Gait Features in Recognizing Depression

**DOI:** 10.3389/fpsyt.2021.661213

**Published:** 2021-05-07

**Authors:** Yameng Wang, Jingying Wang, Xiaoqian Liu, Tingshao Zhu

**Affiliations:** ^1^Chinese Academy of Sciences Key Laboratory of Behavioral Science, Institute of Psychology, Chinese Academy of Sciences, Beijing, China; ^2^School of Computer Science and Technology, University of Chinese Academy of Sciences, Beijing, China; ^3^School of Optometry, The Hong Kong Polytechnic University, Kowloon, Hong Kong; ^4^Department of Psychology, University of Chinese Academy of Sciences, Beijing, China

**Keywords:** depression, gait analysis, machine learning, diagnosis, skeletal joints

## Abstract

While depression is one of the most common mental disorders affecting more than 300 million people across the world, it is often left undiagnosed. This paper investigated the association between depression and gait characteristics with the aim to assist in diagnosing depression. Our dataset consisted of 121 healthy people and 126 patients with depression who diagnosed by psychiatrists according to the Diagnostic and Statistical Manual of Mental Disorders. Spatiotemporal, temporal-domain, and frequency-domain features were extracted based on the walking data of 247 participants recorded by Microsoft Kinect (Version 2). Multiple logistic regression was used to analyze the variance of spatiotemporal (12.55%), time-domain (58.36%), and frequency-domain features (60.71%) on recognizing depression based on Nagelkerke's *R*^2^ measure, respectively. The contributions of the different types of features were further explored by building machine learning models by using support vector machine algorithm. All the combinations of the three types of gait features were used as training data of machine learning models, respectively. The results showed that the model trained using only time- and frequency-domain features demonstrated the same best performance compared to the model trained using all the features (sensitivity = 0.94, specificity = 0.91, and AUC = 0.93). These results indicated that depression could be effectively recognized through gait analysis. This approach is a step forward toward developing low-cost, non-intrusive solutions for real-time depression recognition.

## Introduction

Depression is one of the most common mental disorders affecting more than 300 million people across the world (1). It is associated with decreased life satisfaction, impaired psychosocial functioning, and high disability and suicide rates (2–4). Early treatment can reduce healthcare costs, as well as morbidity and mortality rates associated with depression (5, 6). However, more than half of depressed patients actually have not received treatment for various reasons such as the difficulty in diagnosing (7, 8). One reason mentioned a lot is that primary care physicians generally initiate guideline-concordant care for depressed patients requesting help (9, 10), but they often fail to recognize patients with depressive symptoms (11, 12). Another long-standing reason is traditional questionnaire-based approaches for depression which may increase the risk of misdiagnosing and thus mistreating depression in primary care settings (13–15). Therefore, more efficient methods for detecting depression are required to improve the delivery of services to those in need.

Motor symptoms (e.g., gait) have been shown to be an essential manifestation of depression (16–18). In particular, gait and postural control modulated by a complex neural network (19), which is also implicated with the pathophysiology of major depression (20). Till now, many studies investigated the association between depression and gait characteristics by using instrumental assessments. For example, Sloman et al. (21) analyzed photograms of a single stride during a natural walk and found that depressed patients' walks were more slowly with a lifting motion of the leg. In contrast, healthy participants propel themselves forward with increased foot push-off. Another study used a combination of electronic walkways and photogrammetry to show that depressed patients have shorter strides and slower gait velocity than healthy controls (22). Michalak et al. (23) demonstrated that depressed individuals exhibit reduced vertical head movements, more slumped posture, and lower gait velocity than controls by using three-dimensional (3D) motion capture.

While many studies have demonstrated significant differences in gait patterns between depressed and healthy individuals, gait is relatively neglected in clinical practice as a tool for diagnosing depression (24). For instance, basic clinical gait assessments are mainly observational or based on gait speed to functional assessment (25–27). Nevertheless, judgments formulated by clinicians on the basis of observed behavior are subjective. Methodologies of gait speed tests vary widely from study to study, making it difficult to obtain a general description of patients' gait patterns with depression (28). Furthermore, until recently, automated gait analysis has been requiring expensive equipment and auxiliary operations often unavailable in clinical settings (29). However, modern cost-effective intelligent devices provide new perspectives for gait-based depression recognition. For example, Microsoft Kinect, designed for Xbox, has been used to monitor body movement patterns continuously (30), and its effectiveness in estimating body posture and movement has been proven (29, 31, 32). Using fast Fourier transforms, Zhao et al. (33) extracted frequency domain features from gait data captured by Kinect from 179 graduate students. They then trained machine learning models to predict depression levels of the participants estimated through a questionnaire; the correlation coefficient between the prediction score of models and questionnaire scores reached 0.51. A random forest classifier built based on 12 spatiotemporal features (e.g., walking speed, stride length, arm swing, and body sway) for detecting depression among postgraduate students achieved an accuracy rate 91.58% (34). Wang et al. (35) first extracted time- and frequency-domain features through power spectral density analysis, and spatial geometric features through covariance matrices and the symmetric Stein divergence from gait data captured by Kinect. A framework for detecting depression based on fused features was proposed with a classification accuracy of 93.75%. Therefore, features extracted from Kinect-captured gait data using mathematical methods are effective in model-based depression recognition.

Previous studies showed that machine learning models trained with gait-related features can predict depression accurately, and Kinect provided objective and easily accessible data. However, few of these studies quantified the contribution of each type of gait feature (e.g., spatiotemporal, time-domain, and frequency-domain features) on depression recognition. More importantly, depression severity in these studies was assessed based on depression symptoms scales. While the effectiveness of questionnaire-based scales in accessing depression severity has been well-validated (36), questionnaire scores themselves cannot be used as a diagnosis. Furthermore, research results obtained based on one scale may not replicate to other scales since symptoms on different depression scales do not overlap completely (24).

Instead of considering depressive symptoms in the general population, this study considers the relationship between gait characteristics and depressive symptoms of clinical cases. The aims of the study include (1) evaluating the effect of different types of gait features in recognizing depression and (2) building machine learning models consisting of gait features for detecting depression. Once the contribution and effectiveness of different types of gait features in recognizing depression have been quantified, future research can confidently explore deeper insights into gait patterns of depressed patients and more robust classification models for diagnosing depression in the clinical setting.

## Materials and Methods

### Participants

In this study, depressive patients were recruited from Beijing Anding Hospitals of Capital Medical University. Psychiatrists diagnosed these patients according to the Diagnostic and Statistical Manual of Mental Disorders (DSM-IV) criteria. Inclusion criteria for the case group included: (a) diagnosed as major depressive disorder, (b) no psychotropic medicines are taken within past 2 weeks, (c) without a current or historical DSM-IV diagnosis of any other mental diseases, (d) without current or historical DSM-IV diagnosis of alcohol or drug abuse, and (e) without disability or injury that affected their walking ability.

For the control group, healthy people were recruited via local advertisements. Inclusion criteria for the control group included: (a) both mentally and physically healthy, (b) without long-term use of analgesics, sedatives, sleep drugs, cortisol drugs, anti-epileptic drugs, and treatment of high blood pressure, (c) without positive family history of mental disorders in three generations, and (d) without disability or injury that affected their walking ability.

Finally, 126 depressive patients and 121 healthy people completed the study. Table 1 shows the demographic characteristics of depressed people and healthy people.

**Table 1:** Demographic characteristics of participants.

**Characteristic**	**Depressed group**	**Control group**
**Gender**, ***n*** **(%)**		
Male	57 (45.2)	61 (50.4)
Female	69 (54.8)	60 (49.6)
**Height, M** **±** **SD**	167.4 ± 7.9	166.4 ± 8.2
**Weight, M** **±** **SD**	63.3 ± 11.5	66.4 ± 13.3
**Age, M** **±** **SD**	31.0 ± 9.8	34.7 ± 11.5

### Experimental Settings

**Figure 1:**
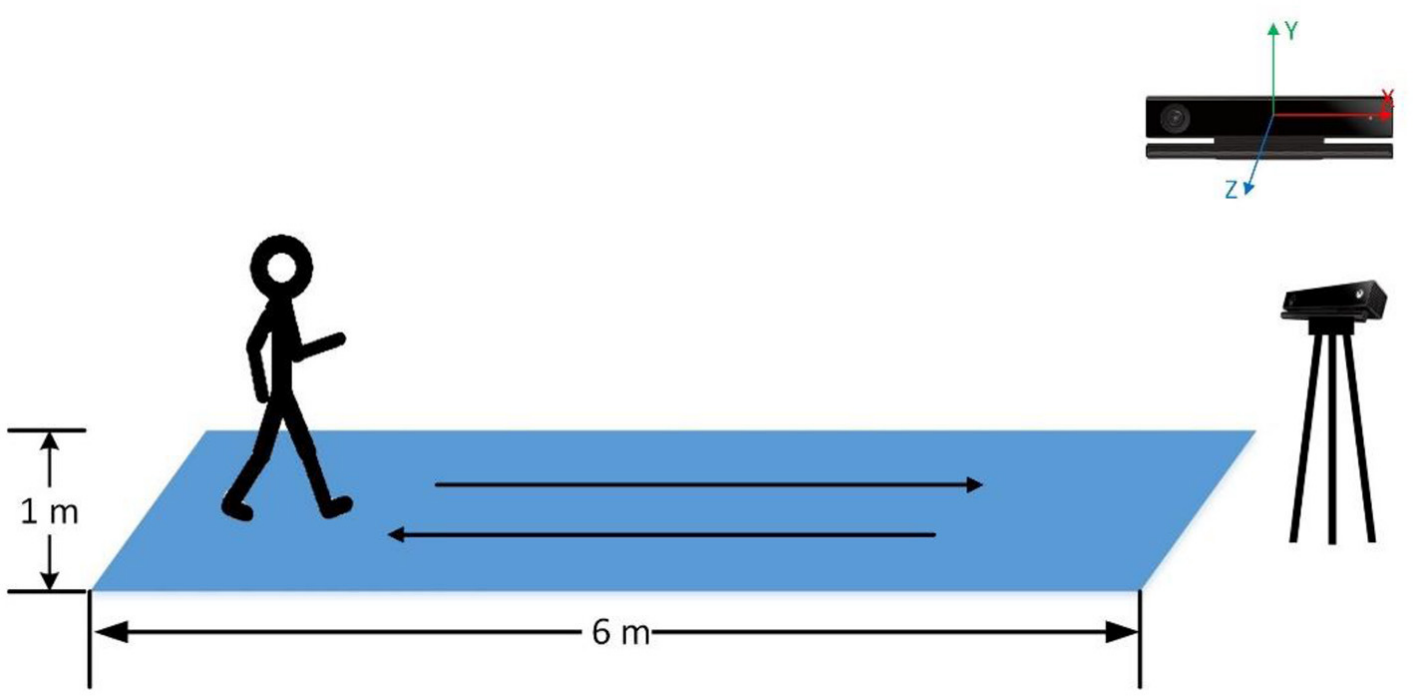
The schematic of the experiment environment.

**Figure 2:**
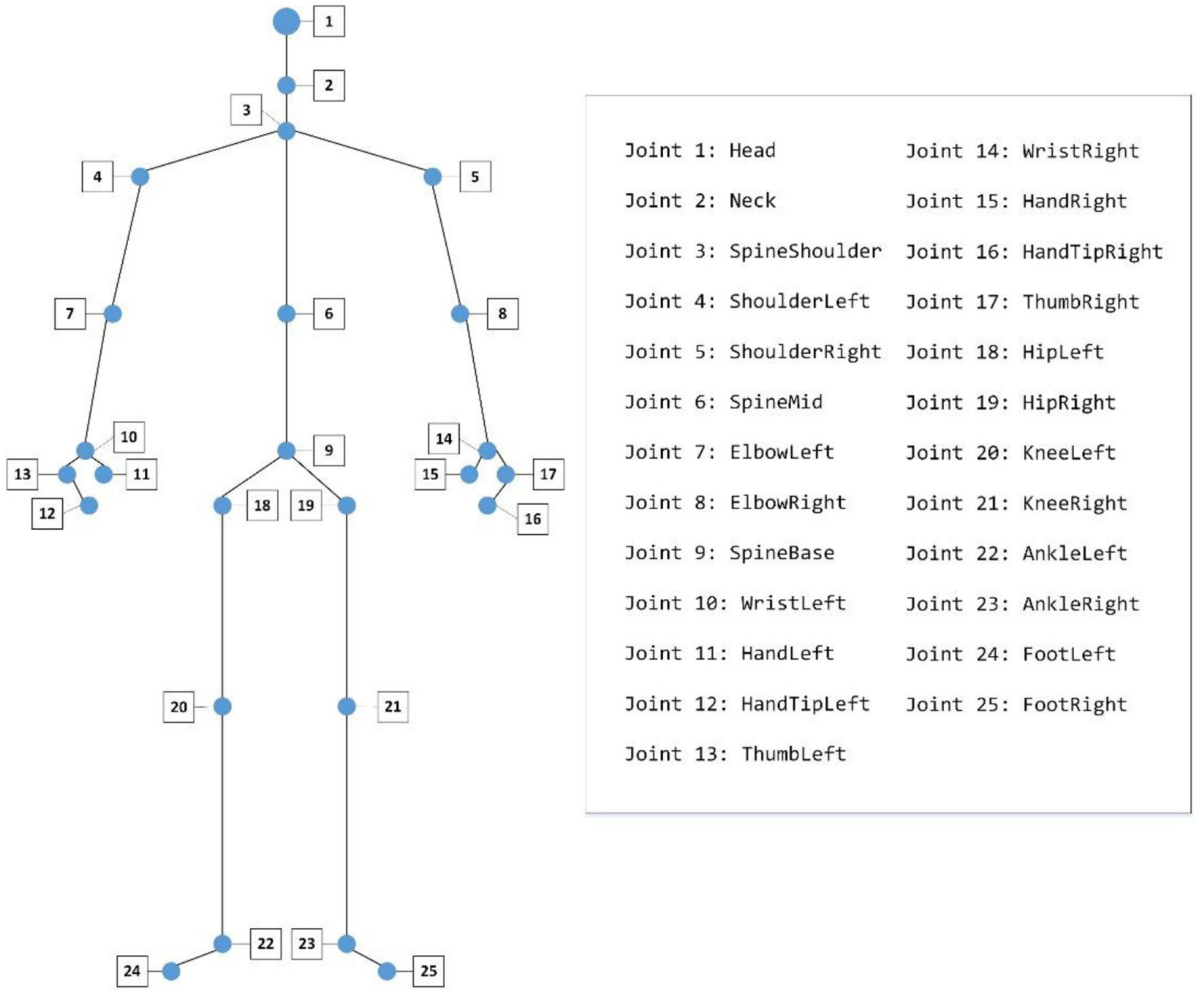
The 25 joints captured by a Kinect.

We used a Kinect (Version 2) to record the gait data of participants. All the participants were asked to walk naturally back and forth for 2 min on a 6 × 1 m footpath (Figure 1). With Kinect continuously shooting, the 3-dimensional position changes of participants' 25 main body joints during walking were recorded by 30 Hz sampling rate (Figure 2). The study was a part of a clinical research project about the potential biological and behavioral indicators of major depressive disorder, approved by the Institutional Review Board of the Institute of Psychology, Chinese Academy of Sciences (approved number: H15010).

### Data Preprocessing

#### Coordinate System Transformation

**Figure 3:**
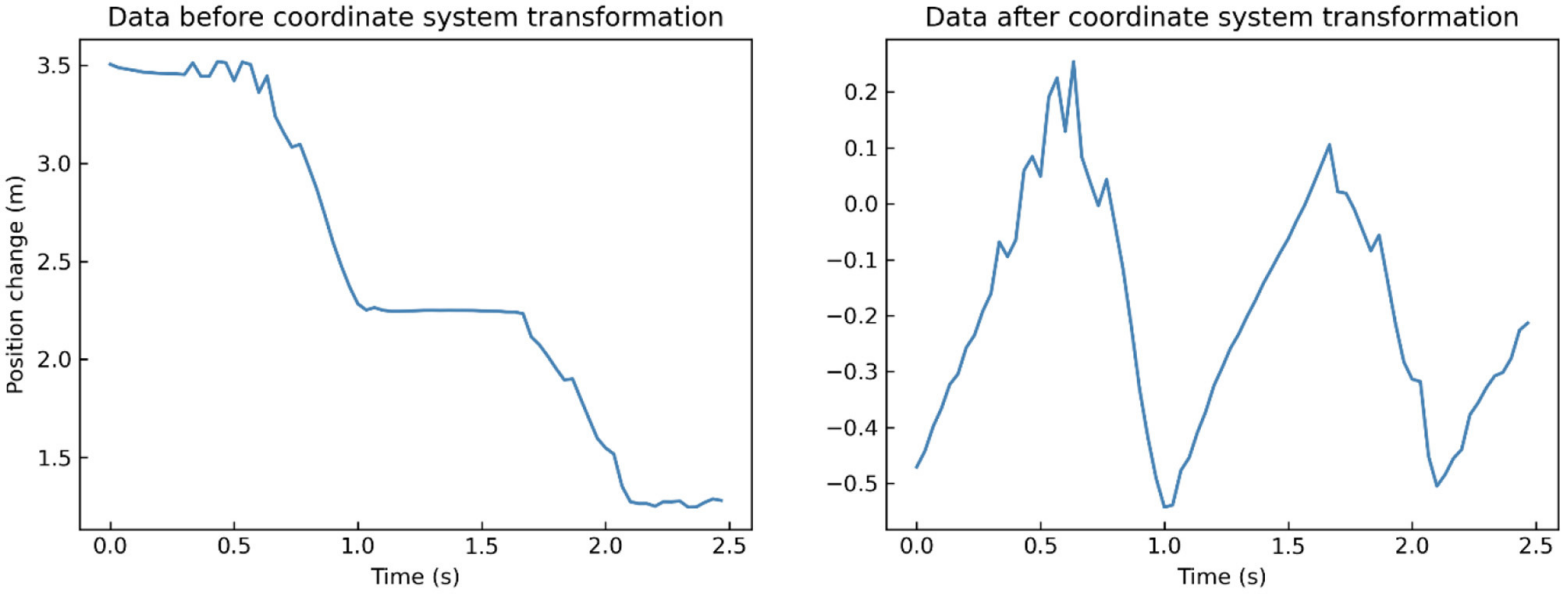
A segment of one participant's left-foot (joint 24) data on Y-axis when walking toward Kinect before (**left**) and after (**right**) coordinate system transformation.

By default, Kinect takes its position as the origin of the 3D Cartesian coordinate system, in which the X-axis grows to the Kinect's left, Y-axis grows up, and Z-axis grows out in the direction Kinect is facing (Figure 1). Given that different participants may have different positions relative to the Kinect during their walk, which means that the default coordinate system may introduce significant errors in gait pattern analysis. Therefore, we need the coordinate system transformation, in which we take the position of the spine joint (joint 9) as the origin of the coordinate system. Taking a segment of one participant's left-foot (joint 24) data on the Y-axis when walking toward Kinect as an example, we demonstrate the effect of the coordinate system transformation (Figure 3).

#### Data Segmentation

Since gait is a cyclic physical activity, large amounts of repetitive data may lead to data redundancy and low computational efficiency. Since each participant's gait data contained back and forth walking, we need to do data segmentation. To do so, we first divided these data into face-toward and back-toward segments based on whether the participant was facing the Kinect or not. We only retained the face-toward segments due to the better accuracy of Kinect in estimating body posture and movement. In order to reduce the influence of participants' unnatural movements at the beginning/end of this experiment, we then selected the middle segment from all the face-toward segments for further analysis. Finally, we identified the beginning of a gait cycle as the lift (toe-off) of left-foot, and chose gait records of two cycles in the middle face-toward segment as the final data segment according to the change of the left-foot (joint 24) on the Y-axis. The length of these segments ranged from 1.73 to 3.43 s (mean = 2.37, SD = 0.26).

#### Low-Pass Filtering

As unexpected body wobble or the systematic errors of Kinect may introduce high-frequency components and noise into the collected data, the original gait record needs to be filtered before data analysis. Gaussian filter is a non-uniform low-pass filter with a kernel whose coefficients decrease with the increase of distance from the kernel's center. It can be used to attenuate noises and high-frequency components in signal data (37), and its effectiveness in filtering Kinect-captured gait data has been proved in several studies (33, 38). Specifically, we set the Gaussian filter's kernel coefficient to $g=\frac{1}{16}\left[1,4,6,4,1\right]$, and then calculated the convolution of each joint' records in 3 dimensions and the Gaussian filter. The procedure of filtering is defined as:

(1) $$\begin{array}{r} \text{y}\left(\text{n}\right)=\sum_{t=-\infty }^{\infty }x\left(t\right)g\left(n-t\right)=x\left(n\right)\;*\;g\left(n\right) \end{array}$$

**Figure 4:**
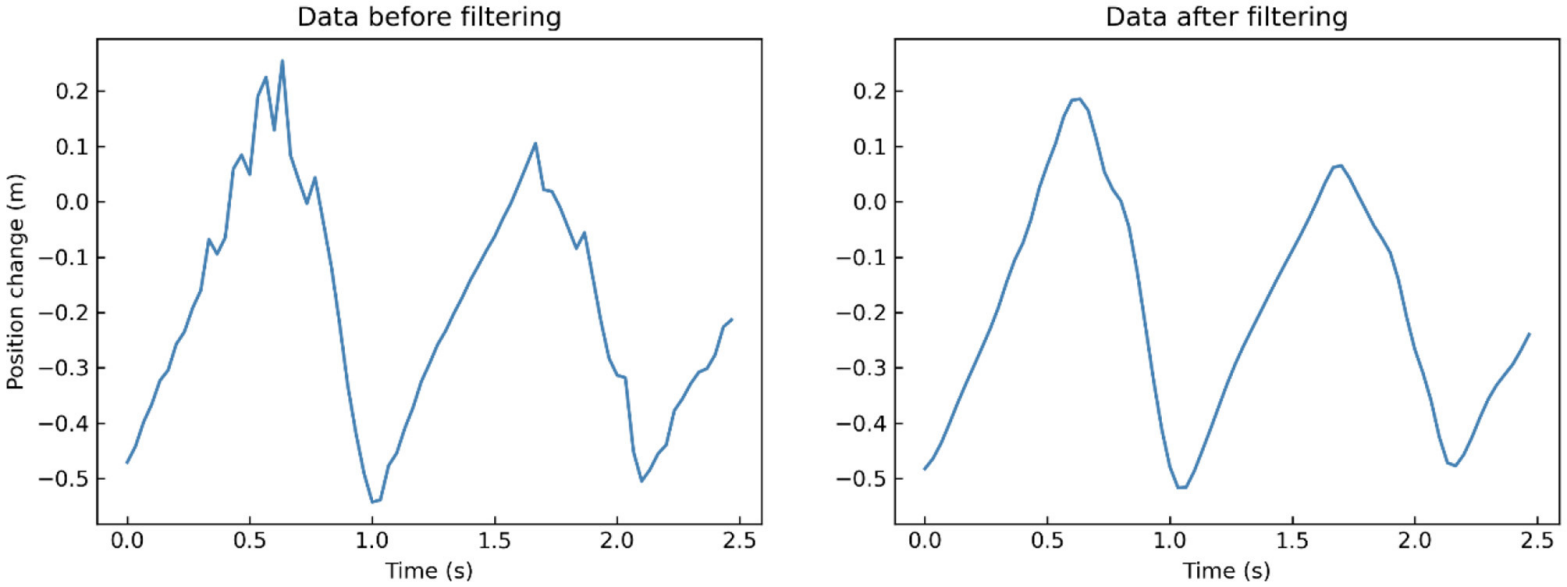
A segment of one participant's left-foot (joint 24) data on Y-axis when walking toward Kinect before (**left**) and after (**right**) low-pass filtering.

*x* is the records of each joint in 3 dimensions, *n* refers to the frame number, *g* refers to the Gaussian filter, and ^*^ refers to convolution operation. We take a segment of one participant's left-foot (joint 24) data on Y-axis when walking toward Kinect as an example. After low-pass filtering, many little burrs and fluctuations in the original records are removed compared to the filtered data (Figure 4).

### Feature Extraction

#### Spatiotemporal Features Extraction

Previous research found that gait patterns associated with depression were characterized by increased body swaying, shortened strides, reduced walking speed and arm swing, etc. (23, 39, 40). Therefore, we extracted spatiotemporal features from Kinect-captured gait data that might potentially differentiate gait patterns between the case and control groups. Specifically, we extracted the following spatiotemporal features:

1) **Body swaying**: Body swaying is measured by the maximum difference in position of the left-shoulder (joint 4) and the right-shoulder (joint 5) on the X-axis during a gait cycle.
2) **Left-arm/Right-arm swing**: It was defined as the maximum difference of the left/right wrist (joint 10/14) moving along the Z-axis during a gait cycle.
3) **Vertical head movement**: We measured the vertical head movement as the maximum vertical amplitude of head (joint 1) along the Y-axis during a gait cycle.
4) **Head posture**: We quantified the head posture during a gait cycle by averaging the angle between the vertical direction and the connection line between the neck (joint 2) and the clavicle (joint 3) in the plane which consisted of the Y-axis and the Z-axis.
5) **Left/Right stride length**: It measured the maximum change in the horizontal direction of the left/right foot (joint 22/23) during a gait cycle.
6) **Left/Right toe clearance**: It measured the maximum change in vertical height of the left/right foot (joint 22/23) during a gait cycle.
7) **Walking speed**: We measured participants' walking speed according to their spine (joint 9) movement along Z-axis.

At last, the mean values of these features in each participant's two gait cycles were calculated as the final spatiotemporal features. Finally, we obtained a total of 10 spatiotemporal features.

#### Time-Domain Features Extraction

Previous studies have confirmed the effectiveness of time-domain features in gait analysis not only in clinical settings but also in laboratory settings (41, 42). Time-domain information related to the statistical value of data on the 25 joints was used to characterize individuals' movement patterns. In this study, we calculated the mean, standard deviation, skewness, and kurtosis of the original data. Specifically, mean is a measure of the central tendency of the random variable characterized by that distribution. Standard deviation measures the amount of dispersion of a dataset. To examine the asymmetry that deviates from the symmetrical bell curve of a dataset, we estimated skewness. Kurtosis measures outliers present in the probability distribution. For the data on the three axes of the 25 joints, we calculated the above four statistical features respectively. Finally, we obtained a total of 3 × 25 × 4 = 300 time-domain features.

#### Frequency-Domain Features Extraction

In addition to using statistical methods to extract time-domain features, we conducted discrete Fourier transform to convert time-domain signals to frequency-domain features (43). The formula is defined as

(2) $$\begin{array}{r} F_{k}=\sum_{j=0}^{n-1}x_{j}^{t}e^{-i2\pi k\frac{j}{n}},\;k=0,1,\ldots ,n-1 \end{array}$$

in which, *i* denotes the imaginary number, $x_{j}^{t}$ stands for data on the *t* axis (*t* ∈ {*X, Y, Z*}), *n* refers to the width of a segment. For the data on the three axes of the 25 joints, we first obtain the amplitudes and phases of the data through the discrete Fourier transform. Then we calculated the direct current component, zero frequency component, which is the average value of the signal, as well as the mean, variance, standard deviation, skewness, and kurtosis of amplitudes and phases, respectively. Finally, we obtained a total of 3 × 25 × (1 + 5 × 2) = 825 frequency-domain features.

### Data Analysis

#### Binary Logistic Regression

The multiple logistic regression analysis was used to investigate the contributing effect of different types of gait characteristics in recognizing depression. In this analysis, the variable selection was performed using stepwise forward selection, subsequently including one by one the variables that were not statistically significant (α = 0.05). Specifically, the dependent variable was composed of dichotomous depressive state (case group vs. control group), and different types of gait features were analyzed as independent variables via multiple logistic regression analysis separately.

Compared with spatiotemporal features, the number of time- and frequency-domain features may be too much, which brings much redundancy and should be filtered out. To avoid the multicollinearity problem and reduce data dimension, principal component analysis (PCA) was initially conducted on the time- and frequency-domain features separately. The principal components (PCs) are the linear combinations of the original features that account for the variance of the data. Then spatiotemporal features, PCs containing 95% cumulative contribution rate of time-domain features, and PCs containing 95% cumulative contribution rate of frequency-domain features entered the logistic regression model.

To measure the contribution effect of variables to depression, we calculated both the odds ratio (OR) (44) and Nagelkerke's *R*^2^ (45). OR is a measure of association between an outcome and exposures. It represents the odds that an outcome will occur given a particular exposure, compared to the odds of the outcome occurring in the absence of that exposure. Nagelkerke's *R*^2^, the adjusted *R*^2^ in linear regression, can provide the amount of variance of the dependent variable explained by the explanatory variables.

#### Classification Modeling

In this stage, we tested the actual classification efficacy of different types of gait features on depression using supervised learning methods, and tried to find the optimal combination of features that makes the depression recognition models have the best performance. Specifically, the output of models was composed of dichotomous depressive state (case group vs. control group). All the combinations of the three types of gait features were used as training data to build machine learning models, respectively. To obtain the optimal performance of machine learning models, we conducted Sequential backward selection (SBS) to remove useless features from training data before building recognition models. SBS is a greedy search algorithm to find the best subset of features, which can minimize the performance loss of machine learning models while reducing the feature dimension (46). It starts from the whole feature set and sequentially discards the feature so as to improve (or minimally worsen) the evaluation measure. The algorithm stops when all remaining features are useful for the model, and removing one of them could lead to a decline in accuracy.

In this study, we trained classification models to detect depression using the support vector machine (SVM) (47) with linear kernel function. SVM is one of the most state-of-the-art classification algorithms, which first maps feature vectors to a higher-dimensional feature space using kernel tricks and then makes predictions based only on support vectors. To evaluate the predictive performance of the models, we considered sensitivity, specificity, and the area under the ROC Curve (AUC) (48). We applied 10-fold cross validation and averaged performance measures across all folds within a single prediction model.

## Results

### Binary Logistic Regression

Results showed that depressed patients walk more slowly (walking speed) and with fewer movements (e.g., left-arm swing, right-arm swing, head posture, right stride length, left toe clearance, right toe clearance) than participants of the control group (Table 2). And the independent sample *t*-test indicated that depressed group had significantly less left-arm swing, right-arm swing, and head posture [*t*_(245)_ = −2.45, *P* = 0.015; *t*_(245)_ = −2.97, *P* = 0.003; *t*_(245)_ = −3.97, *P* < 0.001] than the control group.

**Table 2:** Differences of spatiotemporal features between depressed group and control group.

**Spatiotemporal features**	**Depressed group**		**Control group**		***t* statistic**	***P*-value**
	**Mean**	**SD**	**Mean**	**SD**		
Body swaying (m)	0.36	0.04	0.36	0.04	−0.58	0.562
Left-arm swing (m)	0.27	0.11	0.31	0.12	−2.45	0.015*
Right-arm swing (m)	0.23	0.09	0.27	0.10	−2.97	0.003**
Vertical head movement (m)	0.06	0.05	0.06	0.04	0.42	0.672
Head posture (degree)	1.23	0.10	1.27	0.06	−3.97	<0.001***
Left stride length (m)	0.62	0.07	0.62	0.07	−0.27	0.789
Right stride length (m)	0.59	0.08	0.61	0.07	−1.46	0.146
Left toe clearance (m)	−0.69	0.06	−0.71	0.06	1.92	0.056
Right toe clearance (m)	−0.70	0.06	−0.71	0.07	1.54	0.126
Walking speed (m)	0.99	0.18	1.01	0.17	−0.97	0.332

These spatiotemporal features were calculated in the coordinate system with the spine joint (joint 9) as the origin. *P < 0.05, **P < 0.01, ***P < 0.001.

We examined how many different types of gait features contributed to depression recognition, using Nagelkerke's *R*^2^ statistic, and estimated the effect of each variable using ORs. The results showed that with all the spatiotemporal features in the logistic model, it accounted for 12.55% (Nagelkerke's *R*^2^) of the variance in the dependent variable depression. ORs for each spatiotemporal feature retained in this model after stepwise forward selection and their significance are shown in Table 3; left-arm swing (OR = 0.017, *P* = 0.010), and head posture (OR = 0.001, *P* < 0.001) significantly predicted depression.

**Table 3:** Binary logistic regression model of spatiotemporal features.

	**β**	**SE**	**Wald**	**Odds ratio**	**Corrected *P*-value**
Left-arm swing	−4.047	1.458	7.708	0.017	0.010*
Head posture	−7.031	1.981	12.593	0.001	<0.001***

The P-value is corrected by Bonferroni correction. *P < 0.05, ***P < 0.001.

The results indicated that when all the time-domain PCs entered the logistic model, it accounted for 58.36% (Nagelkerke's *R*^2^) of the variance in the dependent variable depression. ORs for each PC retained in this model after stepwise forward selection and their significance are shown in Table 4; PC2 (OR = 0.740, *P* < 0.001), PC5 (OR = 1.355, *P* < 0.001), PC8 (OR = 1.507, *P* < 0.001), PC11 (OR = 1.253, *P* = 0.037), PC31 (OR = 1.512, *P* = 0.023), and PC35 (OR = 1.926, *P* < 0.001) significantly predicted depression. The time-domain PCs are the linear combinations of the original time-domain features, and Supplementary Table 1 shows the Pearson correlation coefficients between these PCs and the original time-domain features.

**Table 4:** Binary logistic regression model of time-domain principal components.

	**β**	**SE**	**Wald**	**Odds ratio**	**Corrected *P*-value**
PC2	−0.301	0.058	27.001	0.740	<0.001***
PC5	0.304	0.064	22.833	1.355	<0.001***
PC8	0.410	0.077	28.234	1.507	<0.001***
PC9	−0.187	0.74	6.362	0.829	0.198
PC11	0.225	0.073	9.404	1.253	0.037*
PC19	0.231	0.103	5.049	1.260	0.159
PC24	−0.280	0.108	6.752	0.756	0.128
PC26	0.299	0.108	7.631	1.349	0.098
PC28	0.326	0.118	7.580	1.385	0.100
PC29	0.255	0.118	4.658	1.290	0.525
PC31	0.413	0.129	10.257	1.512	0.023*
PC35	0.655	0.156	17.715	1.926	<0.001***
PC39	0.310	0.146	4.502	1.364	0.575
PC41	0.341	0.148	5.291	1.406	0.364
PC46	0.471	0.165	8.200	1.602	0.071
PC70	−0.685	0.248	7.590	0.504	0.100
PC77	−0.709	0.264	7.196	0.492	0.124

The P-value is corrected by Bonferroni correction. *P < 0.05, ***P < 0.001.

The results showed that when all the frequency-domain features PCs entered logistic model, it accounted for 60.71% (Nagelkerke's *R*^2^) of the variance in the dependent variable depression. ORs for each significant PC retained in this model after stepwise forward selection and their significance are shown in Table 5; PC2 (OR = 0.924, *P* = 0.022), PC4 (OR = 1.245, *P* < 0.001), PC5 (OR = 1.278, *P* < 0.001), PC6 (OR = 0.878, *P* = 0.022), PC7 (OR = 0.788, *P* < 0.001), PC10 (OR = 0.855, *P* = 0.024), PC24 (OR = 0.755, *P* = 0.004), PC27 (OR = 0.761, *P* = 0.014), and PC30 (OR = 0.757, *P* = 0.016) significantly predicted depression. The frequency-domain PCs are the linear combinations of the original frequency-domain features, and Supplementary Table 2 shows the Pearson correlation coefficients between these PCs and the original frequency-domain features.

**Table 5:** Binary logistic regression model of frequency-domain principal components.

	**β**	**SE**	**Wald**	**Odds ratio**	**Corrected *P*-value**
PC2	−0.079	0.025	10.349	0.924	0.022*
PC4	0.219	0.043	26.246	1.245	<0.001***
PC5	0.246	0.047	27.014	1.278	<0.001***
PC6	−0.130	0.040	10.370	0.878	0.022*
PC7	−0.238	0.049	23.821	0.788	<0.001***
PC10	−0.157	0.049	10.216	0.855	0.024*
PC11	−0.142	0.052	7.498	0.868	0.105
PC12	0.120	0.055	4.780	1.127	0.490
PC22	0.149	0.070	4.526	1.160	0.568
PC24	−0.281	0.077	13.419	0.755	0.004**
PC27	−0.273	0.081	11.234	0.761	0.014*
PC30	−0.279	0.084	10.900	0.757	0.016*
PC40	0.281	0.098	8.266	1.325	0.069
PC50	0.291	0.115	6.424	1.337	0.191
PC60	0.305	0.125	5.936	1.357	0.252
PC70	−0.355	0.140	6.447	0.701	0.189
PC87	−0.480	0.174	7.604	0.619	0.099

The P-value is corrected by Bonferroni correction. *P < 0.05, **P < 0.01, ***P < 0.001.

### The Recognition of Depression

**Figure 5:**
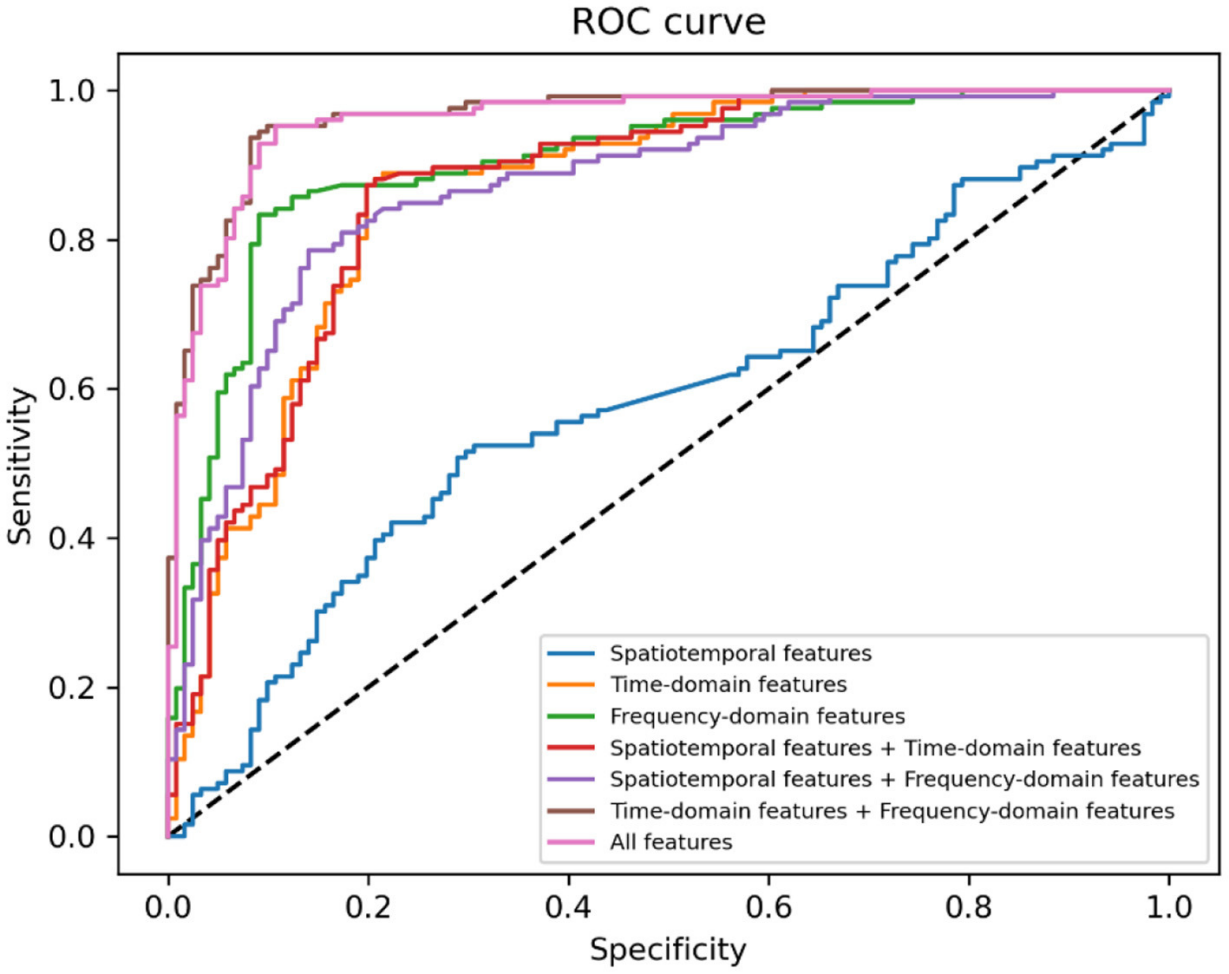
Receiver operating characteristics (ROC) curve for different machine learning methods.

In Table 6, sensitivity, specificity and AUC of the classification models are presented. Figure 5 displays the receiver operating characteristics (ROC) curves of different classification models, showing the balance between sensitivity and specificity throughout the decision space. When only spatiotemporal features were used to classify depression, the classification accuracy (AUC) was 0.58. When only time-domain features were used to classify depression, the classification accuracy (AUC) was 0.83. When only frequency-domain features were used to classify depression, the classification accuracy (AUC) was 0.87. The classification accuracy (AUC) achieved 0.83, when both spatiotemporal features and time-domain features were used to classify depression. The classification accuracy (AUC) achieved 0.83, when both spatiotemporal features and frequency-domain features were used to classify depression. The best accuracy (AUC) is 0.93, when both time- and frequency-domain features or all features were used to classify depression.

**Table 6:** Depression recognition performance measures from 10-fold cross validation.

	**Sensitivity**	**Specificity**	**AUC**
Spatiotemporal features	0.59	0.58	0.58
Time-domain features	0.89	0.78	0.83
Frequency-domain features	0.86	0.88	0.87
Spatiotemporal features + time-domain features	0.89	0.78	0.83
Spatiotemporal features + frequency-domain features	0.82	0.83	0.83
Time-domain features + frequency-domain features	0.94	0.91	0.93
All features	0.94	0.91	0.93

## Discussion

In this study, several machine learning models for detecting depression were trained using gait data captured via Kinect. Ten spatiotemporal features were extracted by following the approaches of previous studies. Besides, 300 time- and 825 frequency-domain features were extracted using time-frequency analysis methods. The results of multiple logistic regression analysis showed that the impacts of spatiotemporal, time-domain, and frequency-domain features on the dependent variable (depression diagnosis) were 12.55%, 58.36%, and 60.71% respectively. The classification models consisting of the above features were found effective in detecting depression. The performance of the optimal model was very outstanding (sensitivity = 0.94, specificity = 0.91, and AUC = 0.93). These findings address the primary goals of the study, which suggest that (1) depression can be reflected in gait, with different types of gait features contributing differently to depression detection and (2) machine learning is an effective approach to recognize depression.

One critical finding in this study is that gait patterns associated with depression are characterized by reduced arm swing and head posture, especially left-arm swing and head posture are predictors of depression in the logistic regression model, which is consistent with previous studies that found that patients with depression tended to walk with reduced movements (e.g., arm swing, head movements) (23, 39, 40). Furthermore, this study validated the effectiveness of both time- and frequency-domain features in recognizing depression, which is also consistent with previous studies (41, 42). However, significant time- and frequency-domain PCs can only provide us with mathematical relationships between gait patterns and time- and frequency- domain features. Although further insights can be gained by calculating the correlation coefficients between each significant PC and original features (as shown in Supplementary Tables 1, 2), they are also not intuitive. It is worth noting that while high-level spatiotemporal features may provide an intuitive understanding of individual gait patterns, they contribute less to depression recognition than low-level time- and frequency- domain features. The limited information contained in spatiotemporal features restricts the possibility of understanding the disorganization of gait control and early detection of gait impairments (49, 50), which is why the clinical gait assessment based on spatiotemporal features (e.g., gait speed) is mainly applicable to monitoring the overall health status of a large population (25).

It is worth noting that the models built of time- domain features and spatiotemporal and time- domain features have the same performances, which suggests that spatiotemporal features had very few contributions to recognize depression. It is also reflected in the fact that the model comprised of time- and frequency-domain features has the same performance as the model consisted of all features. These results are consistent with those reported in previous studies that demonstrated the superiority of signal features such as time- and frequency- domain features over spatiotemporal parameters (42). In summary, time- and frequency-domain features are more efficient in constructing computational models to identify depression than spatiotemporal features.

This study is the first attempt to systematically investigate the impact of different types of gait features on depression recognition. The proposed method allows recognizing depression in real-time and remotely, which can be useful when immediate clinical assessment may not be available. This method is able to overcome many disadvantages of psychological questionnaires (24) include time-consuming (51), recall bias (52), and desirability bias (53). Furthermore, the proposed use of Kinect can be practical in daily-life settings since the devices are low-cost, widely available, and do not require any markers or sensors to be attached to the body. Therefore, the method proposed in this paper holds a promise for detecting depression on a fine-grained scale with ecological validity and low economic burden.

This study has several limitations. First, although Kinect is widely used for estimating body posture and movement (29, 31, 32), it may not record spatiotemporal body data as accurately as more expensive 3D motion capture systems. Second, we could not extract many indicators (e.g., skewness, kurtosis) because the footpath in our experiment is short (6 meters) that resulted in the length of valid gait data we could analyze was short (two cycles) as well. These indicators were computed by high-level spatiotemporal features that could have more contribution to depression recognition if they could be extracted from a longer time gait data. Third, the aim of this study is to examine the contribution of gait features in recognizing depression, thus there is a lot we can optimize to get better model performance (e.g., experimenting with more complex filters, tuning hyper-parameters of machine learning models) from the perspective of engineering.

## Conclusions

This study demonstrated that gait characteristics could be effectively utilized to identify depression, while gait-related features were used for building machine learning models. According to experimental results, spatiotemporal features are appropriate for interpreting gait patterns, while time- and frequency- domain features are effective in depression recognition. In conclusion, the study is a step forward toward developing low-cost, non-intrusive solutions for real-time depression recognition. In the future, this proposed method might be applied in both hospitals to aid diagnosis and scenarios that require a simple and rapid large-scale investigation.

## Data Availability Statement

The datasets generated for this article are not readily available because the raw data cannot be made public, if necessary, feature data can be provided. Requests to access the datasets should be directed to liuxiaoqian@psych.ac.cn.

## Ethics Statement

The studies involving human participants were reviewed and approved by Institutional Review Board of the Institute of Psychology, Chinese Academy of Sciences (approved number: H15010). The patients/participants provided their written informed consent to participate in this study.

## Author Contributions

YW, JW, XL, and TZ contributed to conception and design of the study. YW organized the database and wrote the first draft of the manuscript. YW and JW performed the formal analysis. XL, JW, and TZ contributed to the writing-review and editing of the manuscript. All authors contributed to manuscript revision, read, and approved the submitted version.

## Conflict of Interest

The authors declare that the research was conducted in the absence of any commercial or financial relationships that could be construed as a potential conflict of interest.
